# Human umbilical cord-derived mesenchymal stromal cells protect against premature renal senescence resulting from oxidative stress in rats with acute kidney injury

**DOI:** 10.1186/s13287-017-0475-8

**Published:** 2017-01-28

**Authors:** Camila Eleuterio Rodrigues, José Manuel Condor Capcha, Ana Carolina de Bragança, Talita Rojas Sanches, Priscila Queiroz Gouveia, Patrícia Aparecida Ferreira de Oliveira, Denise Maria Avancini Costa Malheiros, Rildo Aparecido Volpini, Mirela Aparecida Rodrigues Santinho, Bárbara Amélia Aparecida Santana, Rodrigo do Tocantins Calado, Irene de Lourdes Noronha, Lúcia Andrade

**Affiliations:** 1 Division of Nephrology, University of São Paulo School of Medicine, Av. Dr. Arnaldo, 455, 01246-903 São Paulo, Brazil; 2 Department of Internal Medicine, Division of Haematology, University of São Paulo at Ribeirão Preto School of Medicine, Av. Bandeirantes, 3900, 14049-900 Ribeirão Preto, Brazil

**Keywords:** Mesenchymal stromal cell, Acute kidney injury, Senescence, Umbilical cord, Telomere, microRNA

## Abstract

**Background:**

Mesenchymal stromal cells (MSCs) represent an option for the treatment of acute kidney injury (AKI). It is known that young stem cells are better than are aged stem cells at reducing the incidence of the senescent phenotype in the kidneys. The objective of this study was to determine whether AKI leads to premature, stress-induced senescence, as well as whether human umbilical cord-derived MSCs (huMSCs) can prevent ischaemia/reperfusion injury (IRI)-induced renal senescence in rats.

**Methods:**

By clamping both renal arteries for 45 min, we induced IRI in male rats. Six hours later, some rats received 1 × 10^6^ huMSCs or human adipose-derived MSCs (aMSCs) intraperitoneally. Rats were euthanised and studied on post-IRI days 2, 7 and 49.

**Results:**

On post-IRI day 2, the kidneys of huMSC-treated rats showed improved glomerular filtration, better tubular function and higher expression of aquaporin 2, as well as less macrophage infiltration. Senescence-related proteins (β-galactosidase, p21^Waf1/Cip1^, p16^INK4a^ and transforming growth factor beta 1) and microRNAs (miR-29a and miR-34a) were overexpressed after IRI and subsequently downregulated by the treatment. The IRI-induced pro-oxidative state and reduction in Klotho expression were both reversed by the treatment. In comparison with huMSC treatment, the treatment with aMSCs improved renal function to a lesser degree, as well as resulting in a less pronounced increase in the renal expression of Klotho and manganese superoxide dismutase. Treatment with huMSCs ameliorated long-term kidney function after IRI, minimised renal fibrosis, decreased β-galactosidase expression and increased the expression of Klotho.

**Conclusions:**

Our data demonstrate that huMSCs attenuate the inflammatory and oxidative stress responses occurring in AKI, as well as reducing the expression of senescence-related proteins and microRNAs. Our findings broaden perspectives for the treatment of AKI.

**Electronic supplementary material:**

The online version of this article (doi:10.1186/s13287-017-0475-8) contains supplementary material, which is available to authorized users.

## Background

After many cell cycles, the proliferative capacity of a cell decreases because of the loss of telomeres and reduced telomerase activity, a process known as replicative senescence [1]. Other subtypes of senescence, mainly induced by cell stress, have recently been recognised. Stress-induced senescence is classically activated by reactive oxygen species (ROS), and there is evidence that antioxidants delay senescence [2].

In intensive care units, the incidence of acute kidney injury (AKI) can be as high as 10% [3, 4], and ischaemia is a particularly common cause [5] being associated with nearly 30% of all cases of kidney injury in the intensive care unit [6]. Renal ischaemia/reperfusion injury (IRI) can result in a 10–15-fold increase in mortality [7, 8].

In ischaemia-induced AKI, there can be renal production of ROS. Ischaemia/reperfusion stimulates the antioxidant enzyme heme oxygenase-1 (HO-1), responsible for raising the level of manganese superoxide dismutase (MnSOD), a major antioxidant that scavenges superoxide radicals generated within the mitochondria, thereby countering the effects of ROS. However, this process results in cell detachment from tubules, inflammatory cell infiltration and a loss of cell polarity [9], most of the damage to the kidneys being in the tubules. In ischaemia/reperfusion-induced AKI, there are phenotype changes that resemble ageing, such as suppression of the anti-ageing protein Klotho and overexpression of cell-cycle inhibitors [10–12]. The Klotho protein can induce antioxidant enzymes [13], and Klotho deficiency is associated with increased oxidative stress, even in experimental models of kidney disease or in dialysis patients [14].

Mesenchymal stromal cells (MSCs) have been extensively studied in the context of AKI [4, 11–13], mainly because they might act in different pathways that are known to be activated in the pathophysiology of AKI, such as those related to inflammation, apoptosis, angiogenesis and modulation of the immune response [14]. Because of protective mechanisms that appear to involve a paracrine effect via RNA transfer, specifically that of microRNAs (miRs), MSCs constitute a promising treatment for ischaemia/reperfusion-induced AKI [7, 15–17]. Some miRs have been associated with cellular senescence, because they promote redox imbalance and the senescent phenotype [18, 19]. In aged mice treated with bone marrow-derived stem cells, renal markers of senescence have been found to be more numerous among those treated with cells obtained from aged mice than among those treated with cells obtained from young mice [20]. In comparison with adult cells, umbilical cord-derived cells might be more effective in treating AKI. In rat models of AKI, human umbilical cord-derived MSCs (huMSCs) have been shown to repair tissue injury and modulate renal production of ROS [21, 22]. Cells obtained from young mice might present some regenerative factors that those obtained from aged mice do not. Parabiosis studies have shown that, in heterochronic couples, the proliferative and regenerative capacity of cells in aged tissues is improved by contact with younger tissues [5].

The aim of this study was to evaluate the short-term and long-term efficacy of huMSCs in preventing senescence in AKI. To this end, we employed a rat model of ischaemia/reperfusion-induced AKI, in which IRI was followed by no treatment, treatment with huMSCs or treatment with adult human adipose-derived MSCs (aMSCs).

## Methods

### Isolation of huMSCs

Human umbilical cords were collected from healthy infants and mothers at the University Hospital of the University of São Paulo, Brazil. Wharton’s jelly was surgically extracted less than 24 h after delivery. Small (1–5 mm) explants were plated in culture dishes and cultured with alpha-modified Eagle’s medium (Sigma-Aldrich, St. Louis, MO, USA), supplemented with sodium bicarbonate at pH 7.3 (Sigma-Aldrich), penicillin (300 U/ml; Thermo Fisher Scientific, Waltham, MA, USA), streptomycin (300 μg/ml; Thermo Fisher Scientific) and 20% foetal bovine serum (Sigma-Aldrich). Explants were incubated at 37 °C in 5% CO_2_, without changing the medium, for 10–15 days. When cells began to migrate from the explants, the medium was changed every 3–4 days; when they reached 80% confluence, the explants were removed. The cells were then treated with 0.25% trypsin–ethylenediaminetetraacetic acid (trypsin–EDTA; Thermo Fisher Scientific), to be seeded as first-passage (P1) cells. Animals were injected with cells from the third to the fifth passages (P3–P5 cells).

### Isolation of aMSCs

To isolate aMSCs, we collected subcutaneous tissue from an adult patient undergoing elective lipoaspiration surgery. The tissue sample was washed with phosphate-buffered saline (PBS). After digestion with 0.1% collagenase type 1 for 60 min, cells were supplemented with 1% bovine serum albumin and 2 mM CaCl_2_. The stromal fraction was separated by centrifugation at 300 × *g* at room temperature. The aMSCs were cultured and expanded exactly as described for the huMSCs. Animals were injected with P3–P5 cells.

### Cell immunophenotyping

Flow cytometry analysis was performed with allophycocyanin-conjugated, fluorescein isothiocyanate-conjugated, phycoerythrin-cyanine 7-conjugated or phycoerythrin-conjugated antibodies against CD45, CD34, human leukocyte antigen-D region, CD44, CD29, CD105, CD73 and CD90 (BD Biosciences Research, Franklin Lakes, NJ, USA), analysed in a FACSDiva flow cytometer with appropriate software (BD Biosciences Research).

### Lineage differentiation of MSCs

Lineage differentiation was performed with P2 cells seeded in six-well plates at 1 × 10^5^ cells per well. Adipogenesis, osteogenesis and chondrogenesis were tested over periods of 14, 21 and 14 days, respectively, with commercial differentiation kits (StemPro; Thermo Fisher Scientific). Differentiation media were changed every 3–4 days. Adipogenesis, osteogenesis and chondrogenesis were confirmed by staining with Oil Red O, Alizarin and Alcian blue, respectively.

### Cell protein extraction

Total proteins were extracted from P7–P8 huMSCs and aMSCs. The culture medium was aspirated from the adherent cells, which were then washed twice in cold PBS. Radioimmunoprecipitation assay buffer (Sigma-Aldrich) and protease inhibitor cocktail (Sigma-Aldrich) were added, after which the cells were scraped from the plates and sonicated. Samples were centrifuged at 4000 × *g* for 30 min at 4 °C, and supernatants containing total proteins were isolated. Total protein was quantified by bicinchoninic acid protein assay kit (Pierce, Rockford, IL, USA).

### Rat model of renal IRI

Young (2–3 months old) male Wistar–Kyoto rats, weighing 200–300 g, were purchased from the University of São Paulo Biomedical Institute, and all protocols were in accordance with the University of São Paulo Guide for the Care and Use of Laboratory Animals.

**Fig. 1:**
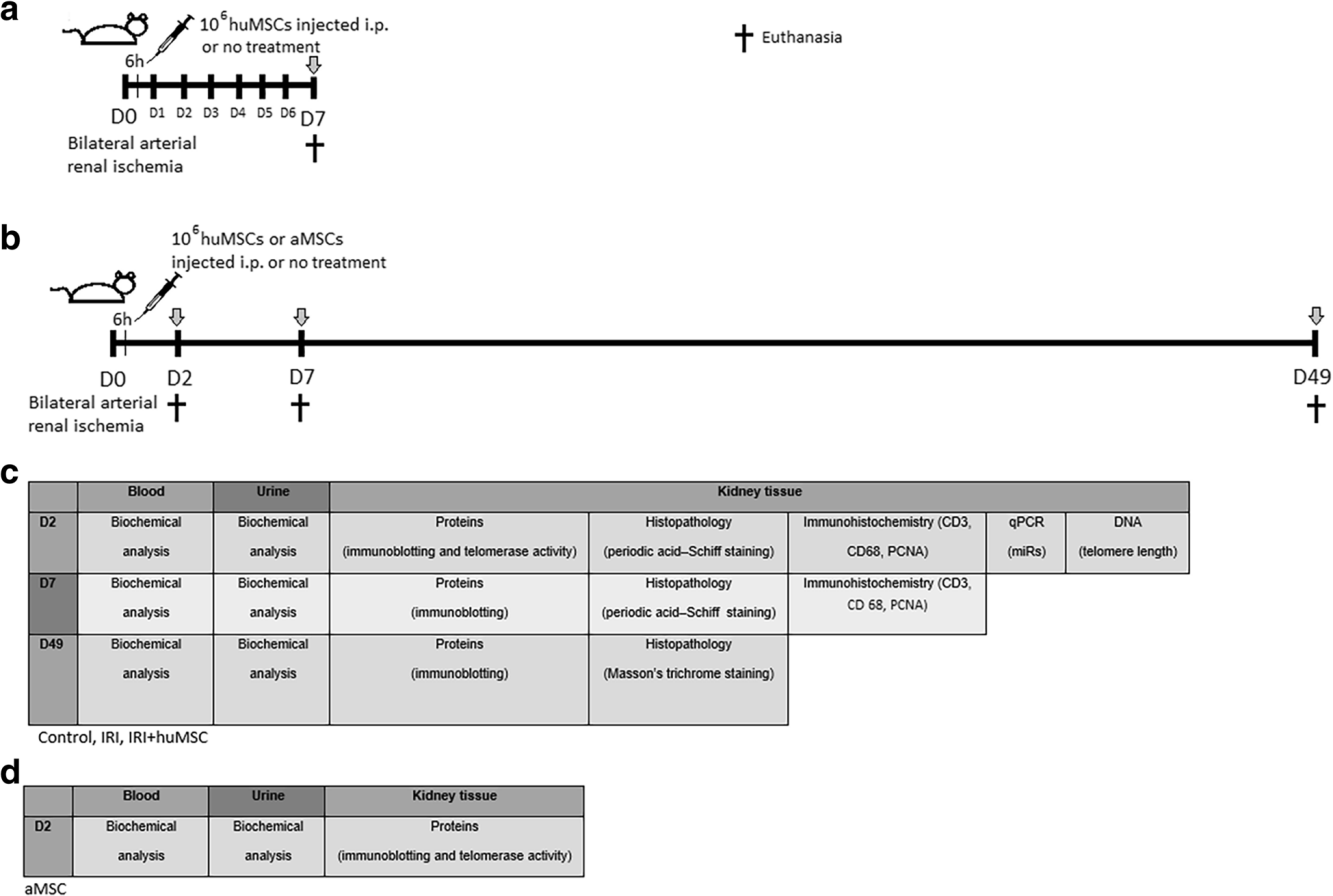
Experimental design. **a** Rats were anesthetised and then submitted to bilateral renal arterial ischemia for 45 min. At 6 h after reperfusion, some rats were injected intraperitoneally with 1 × 10^6^ huMSCs (IRI + huMSC group), whereas others went untreated (IRI group). Data were collected from four control, nine IRI and five IRI + huMSC rats. Plasma urea levels were determined daily over a 7-day period. On day 7, animals were euthanised and blood, urine and kidney samples collected from all. **b** In a second set of experiments, bilateral renal arterial ischemia was induced in 41 rats for 45 min, and after 6 h of reperfusion 20 rats were injected intraperitoneally with 1 × 10^6^ huMSCs (IRI + huMSC group), four rats were injected with 1 × 10^6^ aMSCs (IRI + aMSC group) and 17 rats went untreated (IRI group). On day 2, 22 rats were euthanised (IRI, *n* = 8; IRI + huMSC, *n* = 10; IRI + aMSC, *n* = 4). Twelve rats were euthanised on day 7 (IRI, *n* = 6; IRI + huMSC, *n* = 6), and seven rats were euthanised on day 49 (IRI, *n* = 3; IRI + huMSC, *n* = 4). *Arrows* indicate euthanasia, when blood, urine and kidney samples were collected from all animals. **c** Table indicating the experiments run at each time point in each sample collected from rats in the control, IRI and IRI + huMSC groups. **d** Table indicating the experiments run on day 2 for the IRI + aMSC group. *aMSC* adipose-derived mesenchymal stromal cell, *D2* post-IRI day 2, *D49* post-IRI day 49, *D7* post-IRI day 7, *huMSC* human umbilical cord-derived mesenchymal stromal cell, *IRI* ischaemia/reperfusion injury, *PCNA* proliferating cell nuclear antigen, *qPCR* quantitative polymerase chain reaction

Prior to IRI induction, rats were anesthetised with ketamine (70 mg/kg body weight (BW)) and xylazine (7 mg/kg BW). Both renal arteries were clamped for 45 min. At 6 h after reperfusion, some rats were injected intraperitoneally with 2 ml of saline diluted with 1 × 10^6^ freshly recovered P3–P5 huMSCs (IRI + huMSC group) or 1 × 10^6^ freshly recovered P3–P5 aMSCs (IRI + aMSC group), whereas other rats went untreated (IRI group). To establish the peak of the acute phase and the recovery of renal function, plasma urea levels were determined over a 7-day period (control group, *n* = 4; IRI group, *n* = 9; IRI + huMSC group, *n* = 5). On the basis of the data obtained, we chose to euthanise some animals on post-IRI day 2 (D2) to evaluate the peak, and some animals on D7 to evaluate the recovery. We also euthanised some animals on D49, in order to study the long-term effects on renal function (Fig. 1).

Rats were maintained on a 12-h/12-h light/dark cycle. On D1, D6 and D48, the rats were placed in metabolic cages and 24-h urine samples were collected. On D2, D7 and D49, animals were anaesthetised with ketamine (70 mg/kg BW) and xylazine (7 mg/kg BW), after which arterial blood was collected from the aorta. Animals were then euthanised with an overdose of anaesthesia. Kidneys were flushed with saline and cut into sections: half of the kidneys were fixed in methacarn for histological analysis; the remaining kidneys were stored at −80 °C for further studies.

### Biochemical parameters

We used colourimetric methods to determine plasma creatinine by the Jaffé reaction (Labtest, Brazil) and plasma urea (Labtest, Brazil). To determine plasma sodium we used ion-selective electrodes (AVL 9140 Electrolyte Analyzer; Roche, Basel, Switzerland), and urinary sodium was determined using photometry (CELM, Brazil). Osmolality was measured with a wide-range osmometer (3 W2; Advanced Instruments, Norwood, MA, USA).

### Kidney protein extraction

Kidney samples were homogenised in ice-cold HEPES–KOH buffer, pH 7.5, containing a protease inhibitor cocktail (Sigma-Aldrich) in a homogeniser (PT 10/35; Brinkmann Instruments, Westbury, NY, USA). Homogenates were centrifuged at 4000 × *g* for 30 min at 4 °C to remove nuclei and cell debris. Supernatants, containing total protein, were isolated. To study membrane proteins, the collecting duct water channel aquaporin 2 (AQP2) and Klotho, we performed a second centrifugation of supernatants, at 100,000 × *g* for 1 h at 4 °C, and the pellet was re-suspended in HEPES–KOH to obtain the membrane protein fraction. Total protein and membrane proteins were quantified by bicinchoninic acid protein assay kit (Pierce).

### Electrophoresis and immunoblotting

Kidney and cell samples were run on polyacrylamide gels. Gels were run in duplicate and stained with Coomassie blue (0.1% Coomassie Brilliant Blue R-250, 50% methanol and 10% glacial acetic acid). Selected bands from those gels were scanned, and the density was determined. Gels not so stained were transferred by electroelution to nitrocellulose membranes (Hybond-P; GE Healthcare, Buckinghamshire, UK), and blots were blocked with 5% non-fat dry milk in Tris-buffered saline. Blots were then incubated overnight with antibodies against β-galactosidase (β-gal) at 1:1000 (Sigma-Aldrich); against AQP2 at 1:10,000, Klotho at 1:500, p21^Waf1/Cip1^ (hereafter p21) at 1:500, p16^INK4a^ (hereafter p16) at 1:1000, transforming growth factor beta 1 (TGF-β1) at 1:1000 and Actin at 1:2000 (all Santa Cruz Biotechnology, Dallas, TX, USA); against HO-1 at 1:1000 (Assay Designs, Ann Arbor, MI, USA); and against MnSOD at 1:4000 (Cayman Chemical, Ann Arbor, MI, USA). The labelling was visualised with horseradish peroxidase-conjugated IgG secondary antibody—anti-rabbit (1:2000), anti-mouse (1:2000) or anti-goat (1:10,000) (all Sigma)—and enhanced chemiluminescence detection (Amersham Biosciences; GE Healthcare). Densitometry was used to quantitatively analyse the antibodies, normalising the bands to Actin expression or to selected scanned bands from gels stained with Coomassie Blue (whichever we found to be most suitable for each membrane). Images were visualised with a transilluminator (Alliance 4.2; UVItec, Cambridge, UK) and analysed with ImageJ software (National Institutes of Health, Bethesda, MD, USA).

### Histopathology

Kidneys extracted from animals euthanised on D2 or D7 were processed in paraffin, cut into 4-μm sections and stained with periodic acid–Schiff for light microscopy. The proportional renal damage (tubular epithelial swelling, vacuolar degeneration, necrosis and desquamation) was graded, and a semi-quantitative score of tubular damage was determined as follows: 0, <10%; 1, 10–25%; 2, 26–50%; and 3, >50%.

Kidneys extracted from animals euthanised on D49 were processed in paraffin, cut into 4-μm sections and stained with Masson’s trichrome. A semi-quantitative renal damage score was calculated based on the proportion of the kidney occupied by fibrosis, oedema or inflammatory cells, as follows: 0, none; 1, 1–5%; 2, 6–10%; 3, 10–25%; 4, 26–50%; 5, >50%.

To minimise bias in the morphometric analysis, the observer was blinded to the treatment groups and all microscopic fields were analysed. The mean scores were calculated by rat and by group.

### Immunohistochemistry

We performed immunohistochemical reactions in 4-μm kidney tissue sections from D2 and D7, using antibodies against proliferating cell nuclear antigen (PCNA) and CD3 (1:200 and 1:50, respectively; Dako, Glostrup, Denmark), as well as against CD68 (1:100; Serotec, Hercules, CA, USA). Reaction products were detected by the avidin–biotin–peroxidase complex method (Vector Laboratories, Burlingame, CA, USA), and the colour reaction was developed in 3,3-diaminobenzidine (Sigma-Aldrich) and hydrogen peroxide. Counterstaining was with Harris’ haematoxylin. We analysed 30 juxtamedullary fields. The results of the immunoreactions were quantified by counting the number of positive cells per 0.087-mm^2^ field and averaging the number of cells per field in each section.

### miR analysis

Experiments were performed on kidneys harvested on D2. We extracted miRs using an isolation kit (mirVana; Thermo Fisher Scientific), and we used total RNA enriched with miRs. For reverse-transcriptase reaction, 5 ng of RNA was used, employing the TaqMan MicroRNA Reverse Transcription Kit (Thermo Fisher Scientific). The miRs studied were miR-29a, miR-29b, miR-335 and miR-34a (Applied Biosystems; Thermo Fisher Scientific). RNU48, RNU44, U47 and U6 (Applied Biosystems; Thermo Fisher Scientific) were tested as possible housekeeping genes; we found U6 to be the best suited and used it in our analysis.

To perform a quantitative polymerase chain reaction (qPCR), we used TaqMan Universal PCR Master Mix II (Thermo Fisher Scientific). The data were analysed with DataAssist software (Applied Biosystems; Thermo Fisher Scientific) and relative gene expression calculated as 2^–ΔΔCt^, where Ct is the threshold cycle.

### DNA extraction

Frozen whole kidney tissue digested in proteinase K was used in order to extract genomic DNA with a commercial kit (QIAamp™ DNA mini kit; Qiagen, Venlo, the Netherlands) and the DNA was then quantified using a NanoDrop microvolume spectrophotometer (Thermo Fisher Scientific). The absence of degradation was identified by electrophoresis on an agarose gel containing ethidium bromide.

### Southern blotting to assess telomere length

A 2-mg aliquot of DNA was digested with Fast Digest HinfI (4 μl/2 mg of DNA; Thermo Fisher Scientific) at 37 °C for 2 h. Digested DNA samples were loaded onto 0.8% agarose gels and were run at 80 V for 4 h. To assess telomere length, Southern blotting was performed with a commercial kit (TeloTAGGG Telomere Length Assay; Roche), in which a 5′-TTAGGG-3′ digoxigenin-labelled telomere probe is used and visualised by linking to a chemiluminescent substrate. Blots were imaged in an image analyser (ImageQuant 350; GE Healthcare). We compared two different methods of analysing telomere shortening: the more established method, based on the average telomere lengths (i.e. the mean number of terminal restriction fragments (TRFs)); and a newer method, in which telomeres are stratified by length and the proportions of short (<8.6 kb), medium (8.6–21.2 kb) and long (>21.2 kb) telomeres are determined.

### Telomerase activity

Total kidney protein was used to assess telomerase activity with a commercial kit (TeloTAGGG telomerase PCR ELISA; Roche), which combines PCR and enzyme-linked immunosorbent assay techniques (Additional file 1: Figure S1). To determine the intensity of telomerase activity, we assessed luminescence in a microplate reader (Thermo Fisher Scientific).

### Statistical analysis

Results are expressed as mean ± standard error of the mean. Differences among groups were analysed with GraphPad Prism (GraphPad Software, La Jolla, CA, USA), using analysis of variance followed by Tukey’s post test. When comparisons between two groups were made, unpaired *t* tests were used. When categorical variables were considered, histomorphometry data were also analysed with chi-square tests. Values of *p* ≤ 0.05 were considered significant.

## Results

### Characterisation of huMSCs

**Fig. 2:**
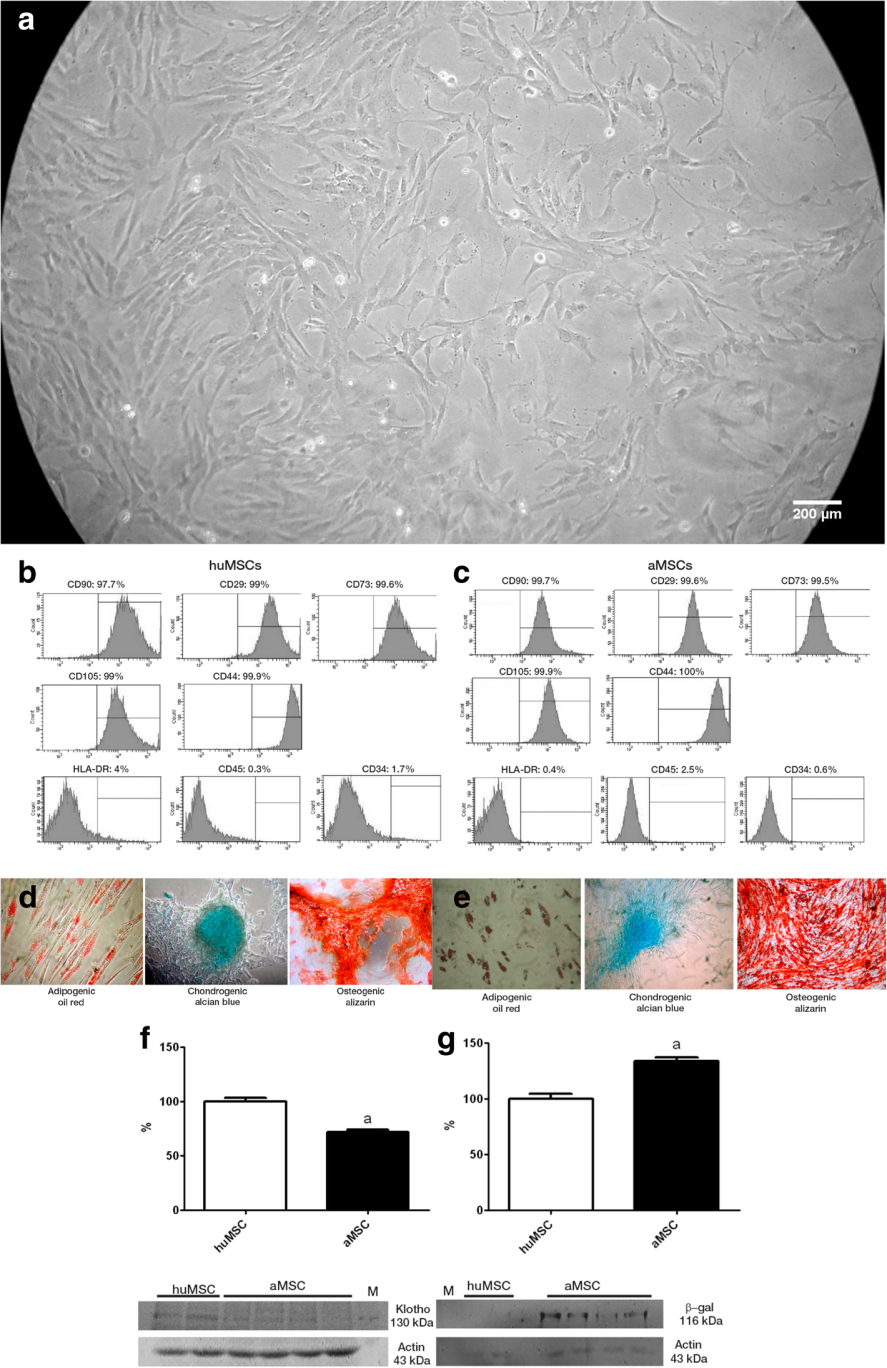
Characterisation of the huMSCs employed. **a** Spindle-shaped cells in cultures of MSCs from Wharton’s jelly. **b** Fluorescence-activated cell immunophenotyping analysis of huMSCs, showing positivity (for CD90, CD29, CD73, CD105 and CD44) and negativity (for human leukocyte antigen-D region (HLA-DR), CD45 and CD34). **c** Fluorescence-activated cell immunophenotyping analysis of aMSCs, showing positivity (for CD90, CD29, CD73, CD105 and CD44) and negativity (for HLA-DR, CD45 and CD34). **d** Analysis of the differentiation capacity of the huMSCs. **e** Analysis of the differentiation capacity of the aMSCs. **f** Klotho: immunoblots and densitometric analysis of samples from huMSCs (*n* = 2) and aMSCs (*n* = 4). **g** β-gal: immunoblots and densitometric analysis of samples from huMSCs (*n* = 2) and aMSCs (*n* = 4). ^a^ *p* < 0.05 vs huMSCs. *aMSC* adipose-derived mesenchymal stromal cell, *huMSC* human umbilical cord-derived mesenchymal stromal cell, *β-gal* β-galactosidase

Figure 2a shows spindle-shaped cells derived from Wharton’s jelly explants of the human umbilical cord, after 2 weeks in culture. We submitted P3–P5 cells to flow cytometry for immunophenotyping. As can be seen in Fig. 2b and c, respectively, huMSCs and aMSCs tested positive for surface markers, including CD90, CD29, CD73, CD105 and CD44, and negative for haematopoietic markers, including human leukocyte antigen-D region, CD45 and CD34. The huMSCs and aMSCs differentiated into adipogenic, chondrogenic and osteogenic lineages (Fig. 2d and e, respectively). Klotho protein expression was higher in huMSCs than in aMSCs (100.0 ± 3.1% vs 71.7 ± 2.1%, *p* < 0.05; Fig. 2f), whereas β-gal expression was lower in huMSCs than in aMSCs (100.0 ± 4.4% vs 133.6 ± 3.4%, *p* < 0.05; Fig. 2g), despite both having been harvested after the same number of passages in culture.

### Treatment with huMSCs ameliorates IRI-induced renal injury

**Fig. 3:**
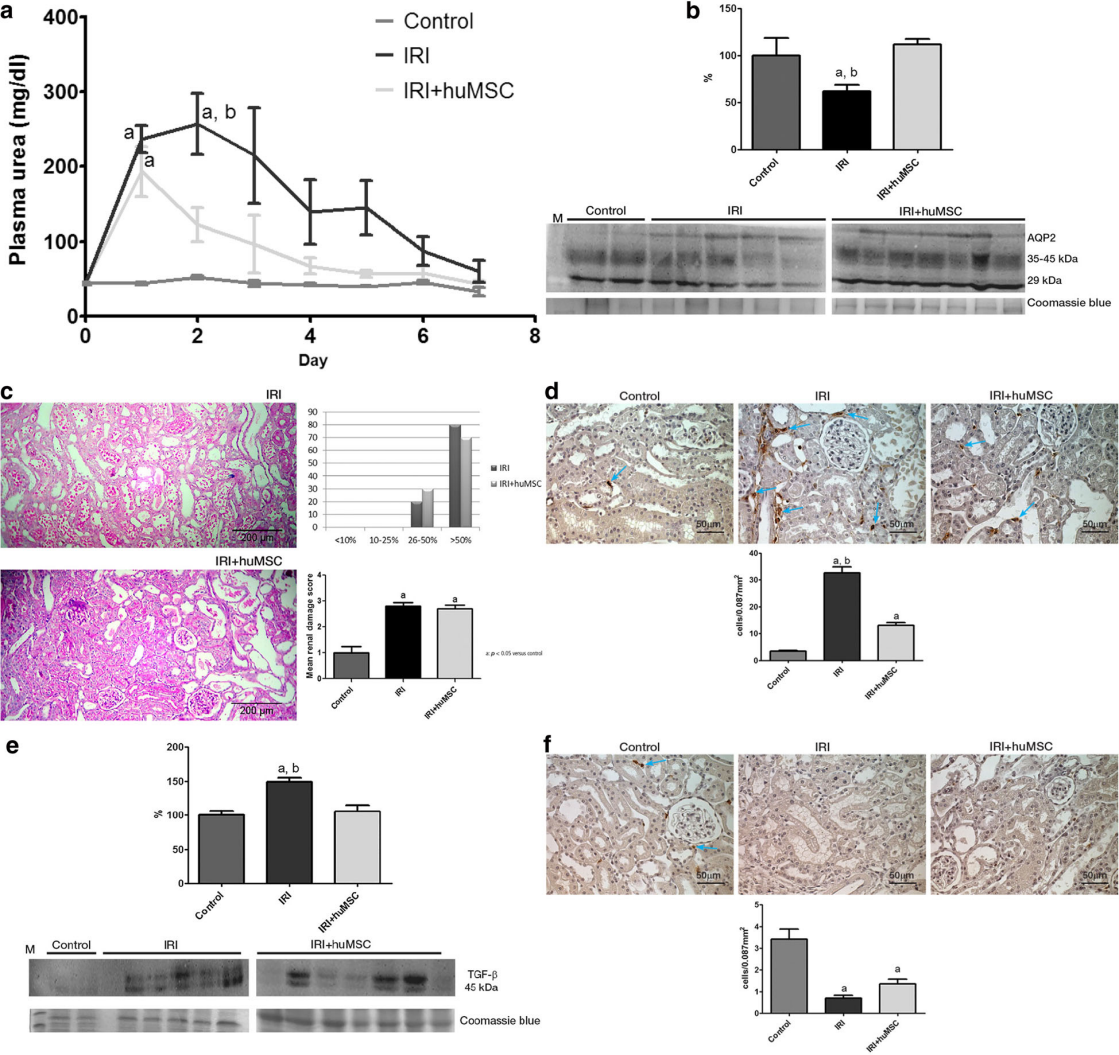
Renal analysis on D2. **a** Plasma urea over time after IRI surgery. **b** AQP2: immunoblots and densitometric analysis of samples from control (*n* = 2), IRI (*n* = 5) and IRI + huMSC (*n* = 7) rats, on D2. **c** Representative light microscopy of periodic acid–Schiff staining and proportional acute tubular damage and mean renal damage scores in IRI and IRI + huMSC rats, on D2 (magnification, ×4). **d** Photomicrographs and bar graphs showing CD68-positive cells (*arrows*) in the tubulointerstitium in control, IRI and IRI + huMSC rats, on D2 (magnification, ×40). **e** TGF-β1: immunoblots and densitometric analysis of samples from control (*n* = 2), IRI (*n* = 5) and IRI + huMSC (*n* = 7) rats, on D2. **f** Photomicrographs and bar graphs showing CD3-positive cells (*arrows*) in the tubulointerstitium in control, IRI and IRI + huMSC rats, on D2 (magnification, ×40). ^a^ *p* < 0.05 vs control. ^b^ *p* < 0.05 vs IRI + huMSC. *huMSC* human umbilical cord-derived mesenchymal stromal cell, *IRI* ischaemia/reperfusion injury, *TGF- β* transforming growth factor beta, *AQP2* aquaporin

Over the 7-day experimental period (D0–D7), plasma urea was consistently lower in IRI + huMSC rats than in IRI rats (Fig. 3a). On post-IRI day 2 (D2), the peaks in plasma urea and creatinine were significantly smaller in the IRI + huMSC group than in the IRI group, whereas creatinine clearance was higher in the former (Table 1). Although creatinine clearance was lower in IRI + huMSC rats than in control rats, the two did not differ in terms of creatinine levels. Therefore, huMSC treatment resulted in partial reversal of the IRI-induced decrease in the glomerular filtration rate.

**Table 1:** Renal function on day 2 after ischaemia/reperfusion

Parameter	Control	IRI	IRI + huMSC
Urea (mg/dl)	52 ± 2.5	234 ± 36.3^a,b^	108 ± 19.6
Creatinine (mg/dl)	0.3 ± 0.02	2.4 ± 0.4^a,b^	0.9 ± 0.17
Creatinine clearance (ml/min/100 g BW)	0.59 ± 0.04	0.10 ± 0.02^a,b^	0.23 ± 0.05^a^
FENa (%)	0.10 ± 0.01	3.27 ± 1.10^a,b^	0.48 ± 0.09
Urinary flow rate (ml/min/100 g BW)	0.002 ± 0.0004	0.005 ± 0.0006^a^	0.006 ± 0.0007^a^

*IRI* ischaemia/reperfusion injury (rats submitted to renal artery clamping for 45 min, followed by 6 h of reperfusion), *IRI + huMSC* IRI + human umbilical cord-derived mesenchymal stromal cells (rats submitted to IRI and subsequently treated with huMSCs), *BW* body weight, *FENa* fractional excretion of sodium

^a^ *p* < 0.05 vs control

^b^ *p* < 0.05 vs IRI + huMSC

On D2, the urinary flow rate was significantly higher in the IRI + huMSC and IRI groups than in the control group (Table 1). The marked IRI-induced increase in urine output was accompanied by a decrease in urine osmolality, which was significantly lower in IRI rats than in control rats (354 ± 24 vs 1231 ± 98 mOsm/kg, *p* < 0.05), although it was significantly higher in IRI + huMSC rats than in IRI rats (450 ± 23 vs 354 ± 24 mOsm/kg, *p* < 0.05). As evidence of the (expected) tubular damage in ischaemia/reperfusion-induced AKI, fractional excretion of sodium (FENa) was higher in the IRI group than in the control group, whereas the IRI + huMSC and control groups showed comparable FENa values (Table 1). As can be seen in Fig. 3b, semi-quantitative immunoblotting revealed that protein expression of AQP2 was lower in IRI rats than in control rats (61.9 ± 7.2% vs 100.1 ± 18.5%, *p* < 0.05). However, AQP2 expression was completely restored in IRI + huMSC rats (112.0 ± 5.6%, *p* < 0.05 vs IRI).

Figure 3c shows the changes in the morphology of renal tubules on D2. At that time point, there was no difference between the IRI and IRI + huMSC groups in terms of the mean renal damage score (2.8 ± 0.13 vs 2.7 ± 0.15).

As depicted in Fig. 3d, the number of cells presenting CD68 staining for macrophages/monocytes in the tubulointerstitium on D2 was significantly higher in the IRI rats than in the control and IRI + huMSC rats (32.6 ± 2.3 vs 3.4 ± 0.3 and 13.1 ± 1.0 cells/0.087 mm^2^, respectively, *p* < 0.05 for both), although the difference between the IRI + huMSC and control rats was also significant (*p* < 0.05). Comparing the IRI and control groups on D2, we found that renal production of the profibrotic protein TGF-β1 was higher in the former (149.5 ± 5.7% vs 100.0 ± 6.2%, *p* < 0.05), although it was normalised (to 105.7 ± 8.8%) in the IRI + huMSC group (*p* < 0.05 vs IRI; Fig. 3e). In addition, the number of cells presenting CD3 staining for lymphocytes in the tubulointerstitium on D2 was significantly lower in IRI and IRI + huMSC rats than in control rats (0.7 ± 0.1 and 1.4 ± 0.2 vs 3.4 ± 0.5 cells/0.087 mm^2^, *p* < 0.05; Fig. 3f).

### Treatment with huMSCs inhibits premature senescence by protecting against the IRI-induced renal pro-oxidative state and cell-cycle inhibition

**Fig. 4:**
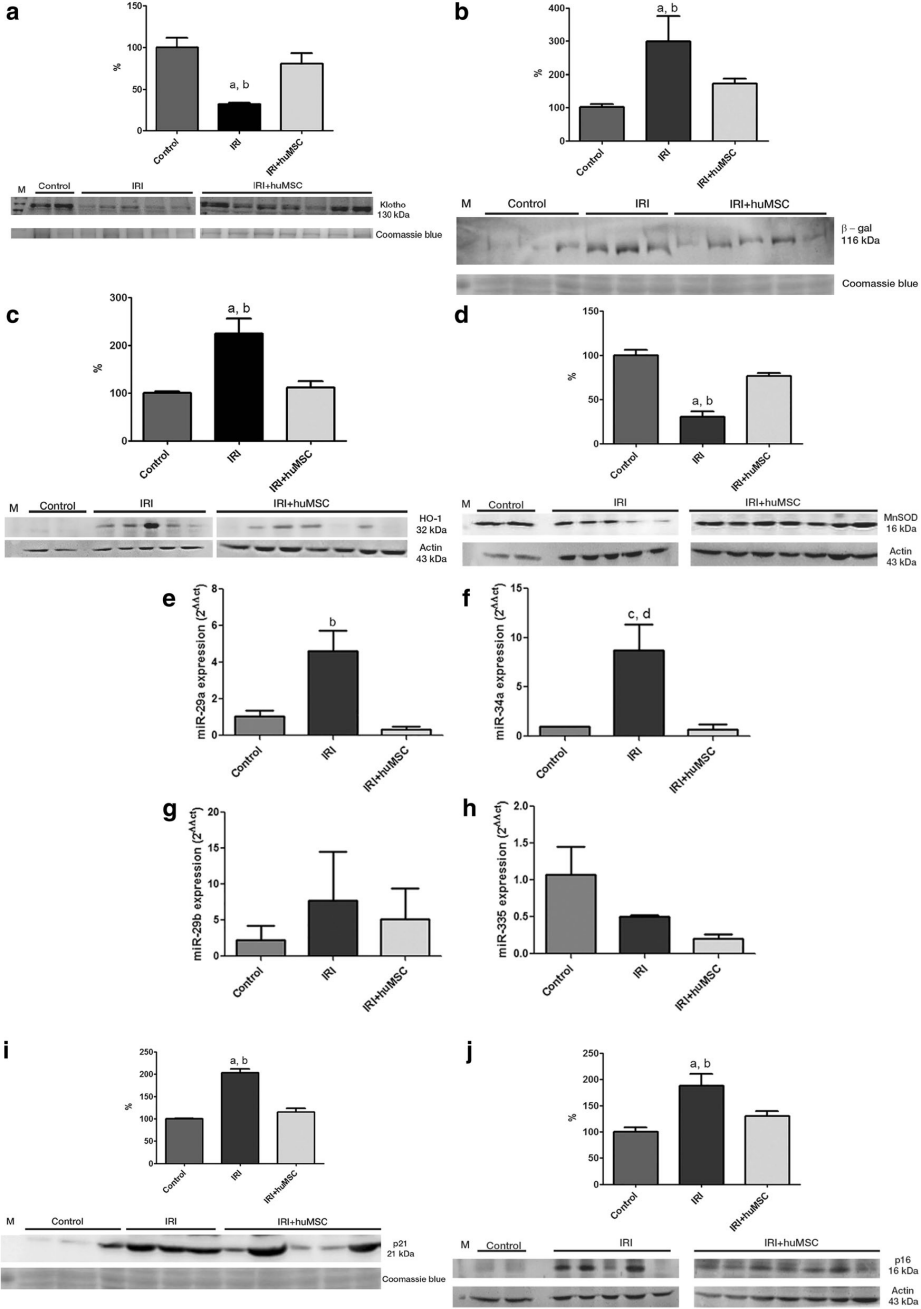
Densitometric analysis and immunoblotting of markers of stress-induced senescence in kidney tissue in control (*n* = 2), IRI (*n* = 5) and IRI + huMSC (*n* = 7) rats, on D2. **a** Immunoblots and densitometric analysis of Klotho. **b** Immunoblots and densitometric analysis of β-gal. **c** Immunoblots and densitometric analysis of HO-1. **d** Immunoblots and densitometric analysis of MnSOD. **e** Bar graphs showing renal miR-29a expression. **f** Bar graphs showing renal miR-34a expression. **g** Bar graphs showing renal miR-29b expression. **h** Bar graphs showing renal miR-335 expression. **i** Immunoblots and densitometric analysis of p21. **j** Immunoblots and densitometric analysis of p16. ^a^ *p* < 0.05 vs control. ^b^ *p* < 0.05 vs IRI + huMSC. ^c^ *p* = 0.05 vs control. ^d^ *p* = 0.05 vs IRI + huMSC. *huMSC* human umbilical cord-derived mesenchymal stromal cell, *IRI* ischaemia/reperfusion injury, *β-gal* β-galactosidase, MnSOD manganese superoxide dismutase, *HO-1* heme oxygenase-1

By D2, Klotho protein expression was dramatically lower in IRI rats than in control rats (32.6 ± 1.8% vs 99.8 ± 12.1%, *p* < 0.05) but was protected in IRI + huMSC rats, in which it was slightly decreased (80.2 ± 13.0%). However, Klotho protein expression did not differ significantly between the IRI + huMSC rats and the control rats (Fig. 4a). By that same time point, the protein expression of β-gal was significantly higher in the IRI group than in the control group (176.7 ± 21.9% vs 97.0 ± 3.3%, *p* < 0.05), although the IRI + huMSC group β-gal protein expression (108 ± 4.2%) was not significantly different from that observed for the control group (Fig. 4b).

On D2, protein expression of HO-1 was higher in IRI rats than in control rats (224.4 ± 32.0% vs 100.0 ± 4.5%, *p* < 0.05), although it remained normal in IRI + huMSC rats (111.8 ± 13.5%; Fig. 4c). In addition, protein expression of MnSOD was lower in IRI rats than in control rats (30.4 ± 6.5% vs 100.0 ± 5.9%, *p* < 0.05), although it was restored in IRI + huMSC rats (111.8 ± 13.5%, *p* < 0.05 vs IRI; Fig. 4d).

Using qPCR, we found that the expression of some senescence-related miRs in kidney tissue (mainly miR-29a and miR-34a; Fig. 4e, f) on D2 was higher in IRI rats than in control rats (4.6 ± 1.1 vs 1.0 ± 0.3 and 8.7 ± 2.7 vs 1.0 ± 0.0, *p* = 0.05) although the expression of both was protected in IRI + huMSC rats (0.3 ± 0.2 and 0.7 ± 0.5, respectively, *p* < 0.05 and *p* = 0.05). Also on D2, the expression of miR-29b and miR-335 (Fig. 4g, h) did not differ significantly among the IRI, IRI + huMSC and control groups: 7.7 ± 6.8, 5.1 ± 4.3 and 2.2 ± 2.0, respectively, for miR-29b; and 0.5 ± 0.0, 0.2 ± 0.1 and 1.1 ± 0.4, respectively, for miR-335.

On D2, expression of the antiproliferative protein p21 was significantly higher in IRI rats than in control rats (202.4 ± 9.2% vs 100.0 ± 1.3%, *p* < 0.05; Fig. 4i). Although elevated p21 expression is expected after IRI, it was not significantly elevated in the IRI + huMSC rats by D2 (115.0 ± 9.1%). Also on D2, expression of the pro-senescence protein p16 was significantly higher in IRI rats than in control rats (187.6 ± 23% vs 100.0 ± 8.6%, *p* < 0.05; Fig. 4j), whereas it remained normal (130.6 ± 8.7%) in the IRI + huMSC rats, the difference between the IRI + huMSC and IRI groups also being significant (*p* < 0.05).

### Ischaemia/reperfusion-induced AKI causes telomere-independent cellular senescence

**Fig. 5:**
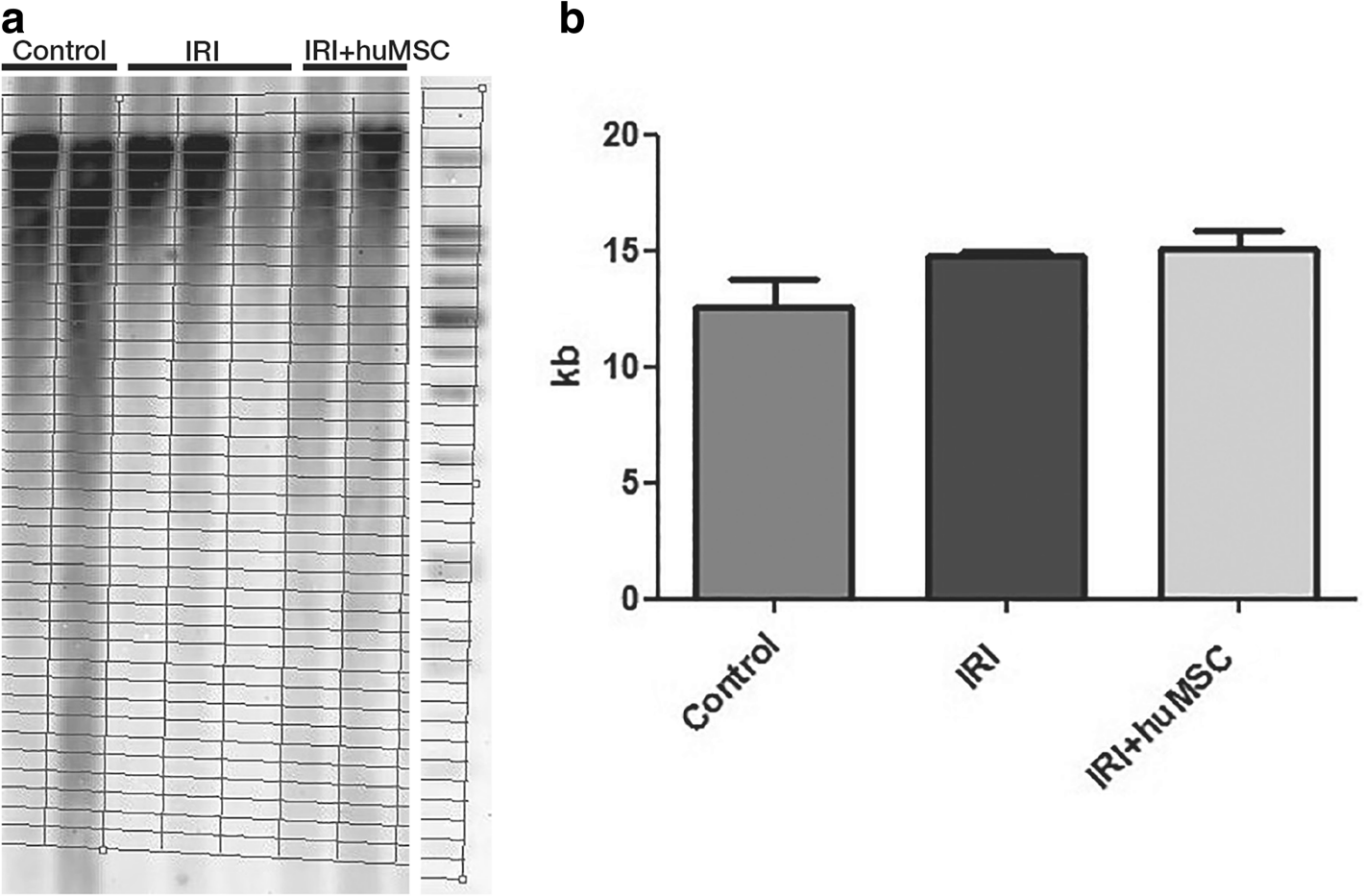
Southern blot and bar graph of telomere lengths. Analysis in control (*n* = 2), IRI (*n* = 3) and IRI + huMSC (*n* = 2) rats on D2. **a** Southern blot plot. **b** TRFs on D2. *huMSC* human umbilical cord-derived mesenchymal stromal cell, *IRI* ischaemia/reperfusion injury

Although markers of senescence were elevated in the rats with ischaemia/reperfusion-induced AKI, the increase did not correlate with telomere shortening. Southern blots of TRFs (Fig. 5a) demonstrated that the mean telomere lengths seen in the IRI and IRI + huMSC groups on D2 (14.7 ± 0.2 and 15.0 ± 0.9, respectively) were comparable with that observed for the control group (12.5 ± 1.2 kb; Fig. 5b). When evaluated in terms of the proportions of short, medium and long telomeres, the groups were also comparable (Table 2). Telomerase activity, as measured by telomeric repeat amplification protocol assay, also did not differ among the groups (data not shown).

**Table 2:** Proportion of long, medium and short telomeres in rat kidneys on D2

Telomere length	Control	IRI	IRI + huMSC
Short telomeres (<8.6 kb) (%)	43.4 ± 9.1	26.9 ± 0.8	26.4 ± 3.2
Medium telomeres (8.6–21.2 kb) (%)	33.5 ± 1.0	42.3 ± 0.6^a^	39.3 ± 0.4^a^
Long telomeres (>21.2 kb) (%)	23.1 ± 8.1	30.8 ± 0.9	34.3 ± 2.8

*IRI* ischaemia/reperfusion injury (rats submitted to renal artery clamping for 45 min, followed by 6 h of reperfusion), *IRI + huMSC* IRI + human umbilical cord-derived mesenchymal stromal cell (rats submitted to IRI and subsequently treated with huMSCs), *kb* kilobase(s)

^a^ *p* < 0.05 vs control

### Treatment with huMSCs does not prevent the IRI-induced increase in PCNA expression in kidney tissue

**Fig. 6:**
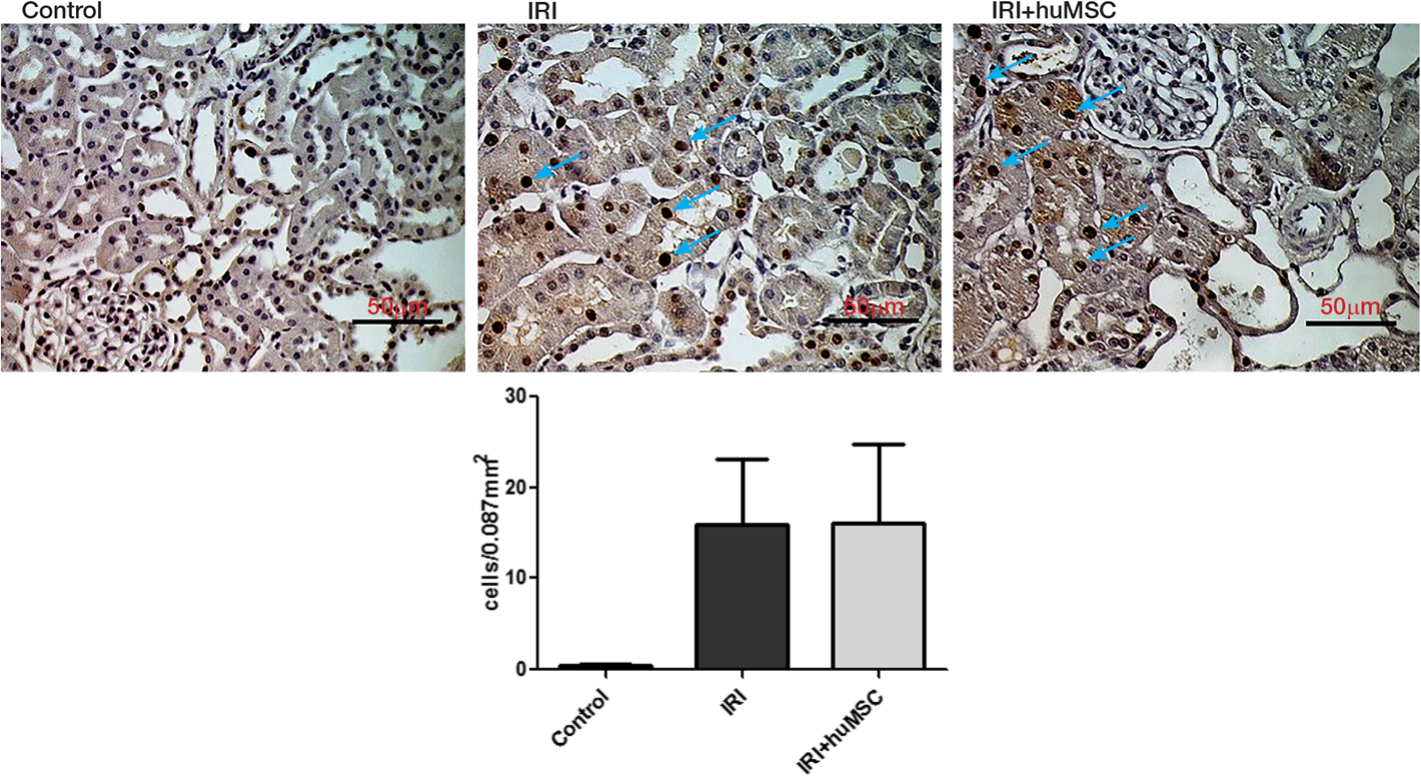
Tubular proliferation. Representative photomicrographs of paraffin-embedded kidney sections stained histochemically for PCNA, with bar graphs showing tubular proliferation in control, IRI and IRI + huMSC rats, on D2. IRI and IRI + huMSC groups differ significantly from control group (*p* < 0.05). *Arrows* indicate positive cells. Magnification, ×40. *huMSC* human umbilical cord-derived mesenchymal stromal cell, *IRI* ischaemia/reperfusion injury

On D2, only a few PCNA-positive cells were seen in control rats (0.5 ± 0.1 cells/0.087 mm^2^). However, the numbers of such cells were elevated in IRI and IRI + huMSC rats (15.9 ± 7.3 and 16.1 ± 8.7 cells/0.087 mm^2^, respectively, p < 0.05 vs control; Fig. 6).

### Treatment with huMSCs protect against maladaptive repair in renal IRI

**Fig. 7:**
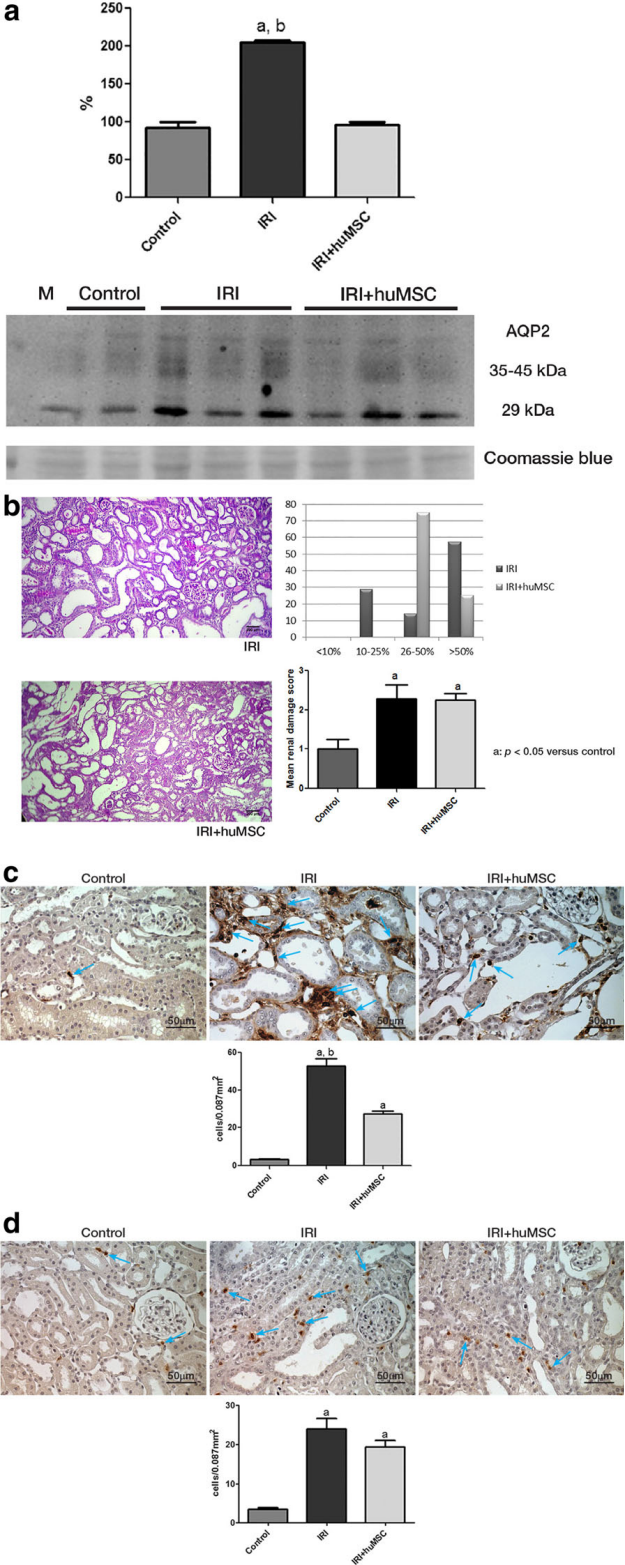
Ischaemia/reperfusion-induced kidney damage on D7. **a** AQP2: immunoblots and densitometric analysis of samples from control (*n* = 2), IRI (*n* = 4) and IRI + huMSC (*n* = 4) rats, on D7. **b** Representative light microscopy of periodic acid–Schiff staining and proportional acute tubular damage and mean renal damage scores in IRI and IRI + huMSC rats, on D7 (magnification, ×4). **c** Photomicrographs and bar graphs showing CD68-positive cells (*arrows*) in the tubulointerstitium in IRI and IRI + huMSC rats, on D7 (magnification, ×40). **d** Photomicrographs and bar graphs showing CD3-positive cells (*arrows*) in the tubulointerstitium in IRI and IRI + huMSC rats, on D7 (magnification, ×40). ^a^ *p* ≤ 0.05 vs control. ^b^ *p* ≤ 0.05 vs IRI + huMSC. *huMSC* human umbilical cord-derived mesenchymal stromal cell, *IRI* ischaemia/reperfusion injury, *AQP2* aquaporin

On D7, there were no statistical differences among the control, IRI and IRI + huMSC groups in terms of the mean plasma urea level (32.6 ± 5.7, 59.8 ± 14.8 and 43.7 ± 4.5 mg/dl, respectively; Fig. 3a) or the mean creatinine level (0.3 ± 0.00, 0.7 ± 0.20 and 0.5 ± 0.2 mg/dl, respectively). Also on D7, IRI rats still presented tubular electrolyte and water handling defects, although IRI + huMSC rats did not. The urinary flow rate was higher in IRI rats than in control and IRI + huMSC rats (0.004 ± 0.0009 vs 0.002 ± 0.0005 and 0.002 ± 0.0003 ml/min/100 g BW, *p* < 0.05), as was FENa (0.38 ± 0.13 vs 0.1 ± 0.01 and 0.14 ± 0.02%, *p* < 0.05). There was no difference between IRI + huMSC rats and control rats in terms of urinary flow rate or FENa. In addition, AQP2 protein expression on D7 did not differ significantly between the IRI + huMSC group and the control group (96.7 ± 3.3 and 92.5 ± 7.5, respectively), although it was significantly higher in the IRI group (205.7 ± 2.3, *p* < 0.05 vs control and IRI + huMSC; Fig. 7a). These data suggest that huMSC treatment counters the effects of IRI, including the urinary concentrating defect and altered renal sodium handling. The mean renal damage score was still high on D7 (2.3 ± 0.36 in IRI vs 2.2 ± 0.16 in IRI + huMSC, *NS*), although most IRI rats presented damage in >50% of the tubular area, compared with 26–50% for the IRI + huMSC rats (Fig. 7b).

On D7, CD68 staining for macrophages/monocytes in kidney tissue was still significantly more pronounced in IRI rats than in control and IRI + huMSC rats (52.9 ± 3.9 vs 3.4 ± 0.3 and 27.3 ± 1.6 cells/0.087 mm^2^, *p* < 0.05), although the significant difference between IRI + huMSC and control rats persisted (*p* < 0.05; Fig. 7c). Despite reduced cells presenting CD3 staining for lymphocytes in the tubulointerstitium on D2, on D7 this number was significantly higher in IRI and IRI + huMSC rats than in control rats (23.9 ± 2.7 and 19.3 ± 1.7 vs 3.4 ± 0.5 cells/0.087 mm^2^, *p* < 0.05; Fig. 7d).

On D7, there were no statistical differences among the groups regarding TGF-β1 expression, oxidative stress or cell-cycle inhibitors (data not shown), except for p16, which was still higher in the IRI group than in the control group (166.7 ± 8.8% vs 105 ± 5%, *p* < 0.05), and stable in the IRI + huMSC group (110 ± 2.9%, *p* < 0.05 vs IRI). By D7, the number of PCNA-positive cells had decreased in the IRI and IRI + huMSC groups (4.0 ± 1.8 and 5.4 ± 3.5 cells/0.087 mm^2^, respectively), the difference between the two being less than significant.

**Fig. 8:**
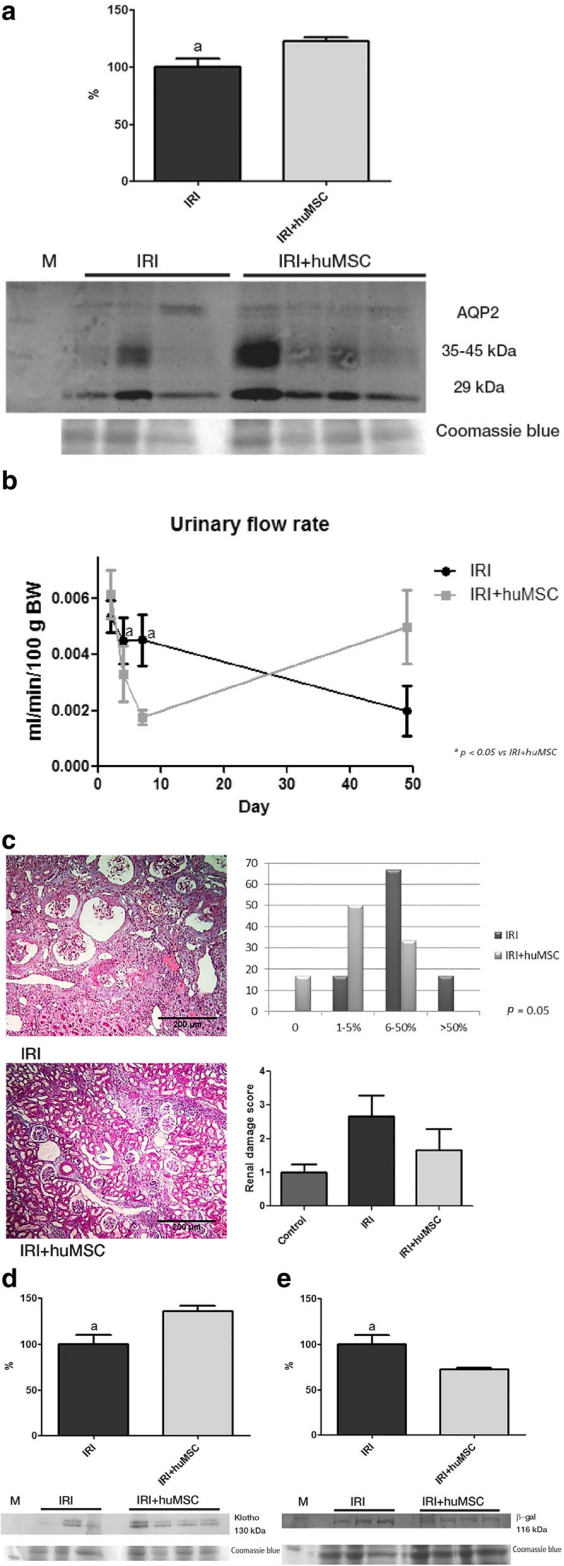
Ischaemia/reperfusion-induced kidney damage on D49. **a** AQP2: immunoblots and densitometric analysis of samples from IRI (*n* = 3) and IRI + huMSC (*n* = 4) rats. **b** Urinary flow rate over the study period. **c** Representative light microscopy of Masson’s trichrome staining and chronic renal damage score in IRI and IRI + huMSC rats (magnification, ×4). **d** Klotho: immunoblots and densitometric analysis of samples from IRI (*n* = 3) and IRI + huMSC (*n* = 4) rats. **e** β-gal: immunoblots and densitometric analysis of samples from IRI (*n* = 3) and IRI + huMSC (*n* = 4) rats. ^a^ *p* ≤ 0.05 vs IRI + huMSC. *huMSC* human umbilical cord-derived mesenchymal stromal cell, *IRI* ischaemia/reperfusion injury, *AQP2* aquaporin, *β-gal* β-galactosidase, *BW* body weight

On D49, plasma urea, creatinine and FENa were significantly higher in IRI rats than in IRI + huMSC rats (Table 3), and the urinary concentrating defect persisted in the former group. Urine osmolality was lower in IRI rats than in IRI + huMSC rats (480 ± 61 vs 513 ± 39 mOsm/kg, *p* < 0.05), as was renal expression of AQP2 (100.0 ± 7.7 vs 122.8 ± 3.3; Fig. 8a). Despite the urinary concentrating defect, the urinary flow rate was comparable between the IRI and IRI + huMSC groups (Fig. 8b). On D49, there was marked renal damage in the IRI rats, as evidenced by the fact that the renal damage score was higher in the IRI group than in the IRI + huMSC group (2.7 ± 0.61 vs 1.7 ± 0.61; Fig. 8c). Also on D49, senescence was more apparent in IRI rats than in IRI + huMSC rats, Klotho expression being significantly lower in the IRI group than in the IRI + huMSC group (100.0 ± 10.0 vs 136.2 ± 5.6, *p* < 0.05; Fig. 8d) and β-gal protein expression being significantly higher in the former (100.0 ± 10.5 vs 72.3 ± 2.0, *p* < 0.05; Fig. 8e). Immunoblotting for TGF-β1, p21 and p16 did not reveal any differences among the groups (data not shown).

**Table 3:** Renal function on day 49 after ischaemia/reperfusion

Parameter	IRI	IRI + huMSC
Urea (mg/dl)	65 ± 26.7^a^	52 ± 5.0
Creatinine (mg/dl)	0.9 ± 0.26^a^	0.6 ± 0.03
Creatinine clearance (ml/min/100 g BW)	0.29 ± 0.08	0.42 ± 0.1
FENa (%)	0.07 ± 0.03^a^	0.03 ± 0.01
Urinary flow rate (ml/min/100 g BW)	0.002 ± 0.0009	0.005 ± 0.0013

*IRI* ischaemia/reperfusion injury (rats submitted to renal artery clamping for 45 min, followed by 6 h of reperfusion), *IRI + huMSC* IRI + human umbilical cord-derived mesenchymal stromal cells (rats submitted to IRI and subsequently treated with huMSCs), *BW* body weight, *FENa* fractional excretion of sodium

^a^ *p* < 0.05 vs IRI + huMSC

### Treatment with huMSCs reduces IRI-induced renal damage to a lesser degree than does treatment with aMSCs

**Fig. 9:**
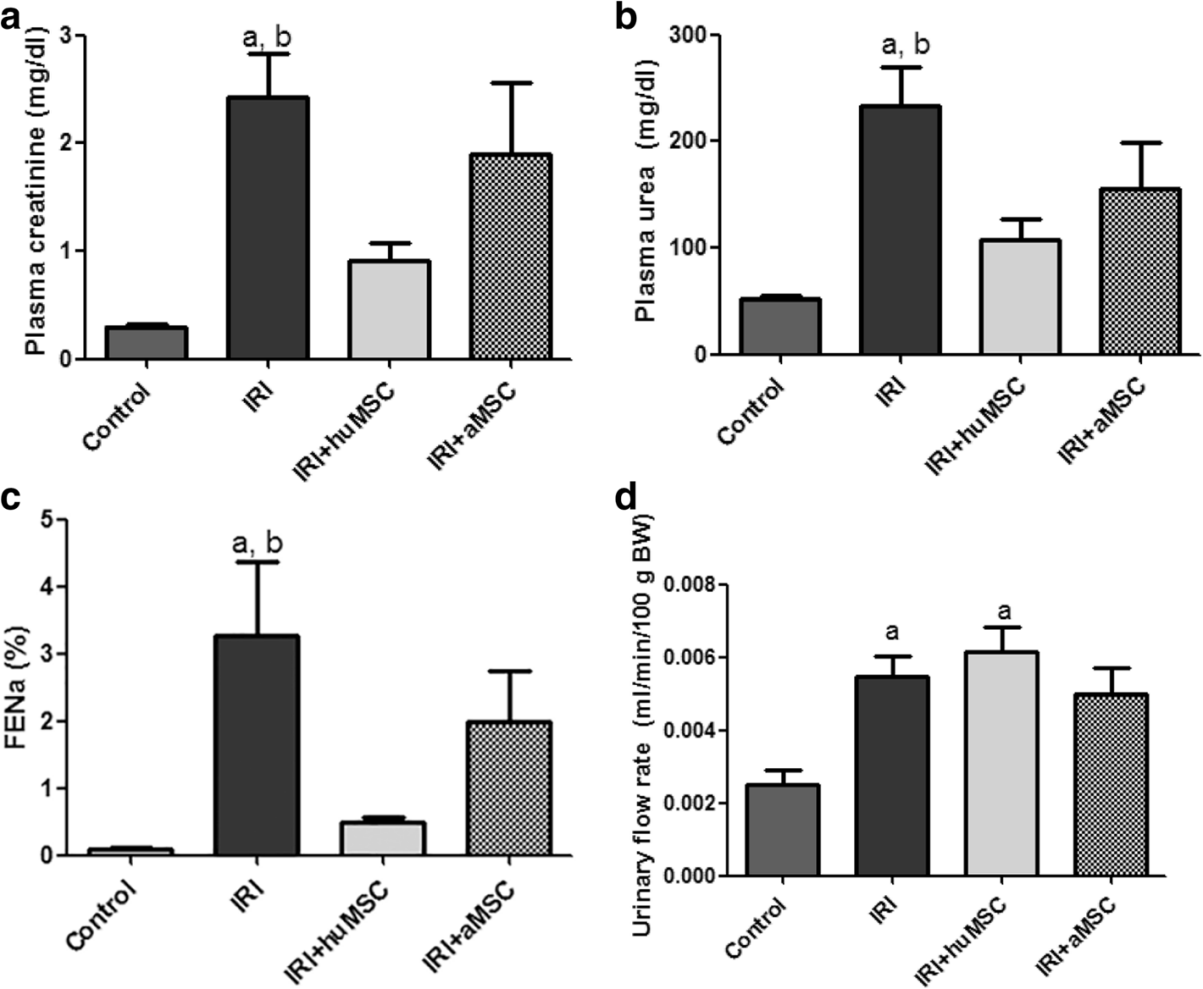
Ischaemia/reperfusion injury treated with huMSC or aMSCs. Bar graphs showing parameters in control, IRI, IRI + huMSC and IRI + aMSC rats, on D2. **a** Plasma creatinine. **b** Plasma urea. **c** FENa. **d** Urinary flow rate. ^a^ *p* < 0.05 vs control. ^b^ *p* < 0.05 vs IRI + huMSC. *aMSC* adipose-derived mesenchymal stromal cell, *huMSC* human umbilical cord-derived mesenchymal stromal cell, *IRI* ischaemia/reperfusion injury, *FENa* fractional excretion of sodium, *BW* body weight

By D2, the treatment with aMSCs had resulted in improved renal function, although the improvement was less pronounced than that observed on D2 for the rats treated with huMSCs. Although plasma creatinine, plasma urea and FENa were lower in the IRI + aMSC group than in the IRI group (1.9 ± 0.66 vs 2.4 ± 0.4 mg/dl, 156 ± 43.3 vs 234 ± 36.3 mg/dl and 1.98 ± 0.76 vs 3.27 ± 1.10%, respectively; Fig. 9a–c), these differences did not reach statistical significance, and they were higher in the IRI + aMSC group than in the IRI + huMSC group. The urinary flow rate in the IRI + aMSC group did not differ significantly from that observed for the IRI and IRI + huMSC groups (0.005 ± 0.0007 vs 0.005 ± 0.0006 and 0.006 ± 0.0007 ml/min/100 g BW; Fig. 9d). In addition, there was no significant difference between the IRI + aMSC and IRI + huMSC groups in terms of the renal expression of AQP2 (95.7 ± 15.8% vs 112.0 ± 5.6%).

**Fig. 10:**
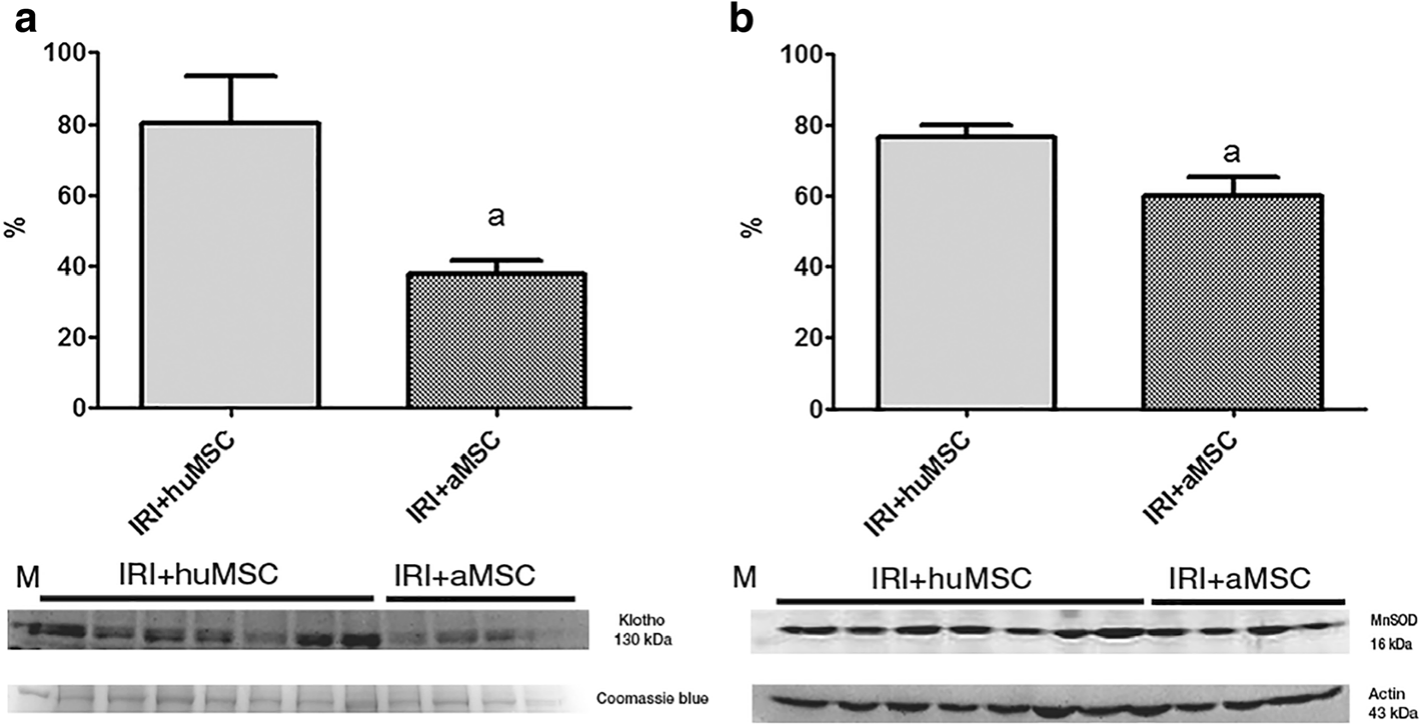
Densitometric analysis and immunoblotting of premature senescence markers that behaved differently in IRI + huMSC rats than in IRI + aMSC rats. Immunoblots and densitometric analysis of samples from IRI + huMSC (*n* = 7) and IRI + aMSC rats (*n* = 4), on D2. **a** Klotho. **b** MnSOD. ^a^ *p* < 0.05 vs IRI + huMSC. *aMSC* adipose-derived mesenchymal stromal cell, *huMSC* human umbilical cord-derived mesenchymal stromal cell, *IRI* ischaemia/reperfusion injury, *MnSOD* manganese superoxide dismutase

Renal expression of Klotho was lower among the animals treated with aMSCs than among those treated with huMSCs (37.6 ± 3.7 vs 80.2 ± 13.0, *p* < 0.05; Fig. 10a). Expression of HO-1 was comparable between the IRI + huMSC and IRI + aMSC groups (111.8 ± 13.5 vs 98.9 ± 2.3%). However, MnSOD expression was lower in the IRI + aMSC group than in the IRI + huMSC group (60.2 ± 5.2 vs 76.6 ± 3.4, *p* < 0.05; Fig. 10b). There were no statistical differences between the IRI + huMSC and IRI + aMSC groups regarding the expression of TGF-β1 or cell-cycle inhibitors.

## Discussion

In the present study, we demonstrated that rats submitted to renal ischaemia for 45 min develop AKI that becomes pronounced by 48 h after IRI, at which point we observed a peak in plasma urea, a drop in the glomerular filtration rate (i.e. creatinine clearance) and acute tubular necrosis, leading to increases in the urinary flow rate and FENa, as well as urinary concentrating defects, mainly due to decreased protein expression of AQP2. At D7, AQP2 expression was higher among the animals subjected to IRI than among the controls, as was the urinary flow rate. In the normal state, urine is concentrated as a result of the combined functions of Henle’s loop and the collecting duct [23]. The increased AQP2 expression following IRI might be a compensatory response to defects in other tubular segments, serving to limit the urinary flow rate. In our long-term analysis, we also found that, despite the fact that urinary osmolality and renal AQP2 expression were lower among the animals subjected to IRI only than among those subjected to IRI and subsequently treated with huMSCs, the urinary flow rate was not higher in the former group, as would have been expected. One possible explanation for that is that the glomerular filtration rate was decreased in the animals that were subjected to IRI and left untreated.

We found that huMSC treatment improved glomerular filtration and tubular function. At 48 h after IRI, huMSC-treated rats presented improved renal function, as well as an improvement in urinary concentrating ability and less macrophage infiltration of kidney tissue. In addition, expression of the p21, p16, TGF-β1 and β-gal pathways, which initiate a senescence response in AKI, was normalised in huMSC-treated animals. We also demonstrated that huMSC treatment provided protection against the IRI-induced decrease in Klotho protein expression, as well as against the oxidative stress seen in AKI. Treatment with huMSCs restored HO-1 to pre-IRI levels and increased protein expression of MnSOD to levels similar to those observed for controls.

It is known that stem cells from young donors are more effective in the treatment of aged kidneys than are those obtained from older donors. In one study, aged mice were exposed to a lethal dose of radiation and then treated via transplantation of bone marrow from age-matched or younger donor mice [20]. The authors found that the recipients of stem cells obtained from older donors showed high renal expression of senescence-related proteins, low Klotho expression and significant macrophage infiltration in the kidneys. In the recipients of stem cells from young donors, the inverse was true for all of these parameters [20], indicating that such treatment is more effective than is treatment with stem cells from older donors. In comparison with MSCs derived from human bone marrow or from adipose tissue, huMSCs have been shown to be more effective in reducing the expression of cell-cycle inhibitors [24]. In the present study, we compared huMSCs with aMSCs in terms of their ability to ameliorate the effects of IRI. We demonstrated that, although the treatment with aMSCs minimised renal damage after IRI, it was less effective than was the treatment with huMSCs in countering the effects of ischaemia/reperfusion-induced AKI. This observation might be related to the fact that Klotho expression was higher in huMSCs than in aMSCs. Klotho might promote healing from renal IRI [12], possibly by inducing MnSOD expression [25] and protecting against a pro-oxidative state, given that Klotho increases the phosphorylation of FOXO3a, a transcription factor that upregulates MnSOD [17, 18]. In fact, the kidneys of animals treated with huMSCs expressed more Klotho and more MnSOD than did those of animals treated with aMSCs.

The IRI pathway involves oxidative imbalance, and Klotho deficiency has been a well-defined hallmark of ischaemia/reperfusion-induced AKI [12, 14]. Commonly, MnSOD inhibits mitochondrial production of ROS and is activated by HO-1 via carbon monoxide production [26, 27]. When renal medullary activity of HO-1 is blocked, there is a decrease in MnSOD expression [28]. In our model of IRI, despite elevated HO-1 expression, MnSOD expression is reduced, resulting in a pro-oxidative state. We can speculate that the normalisation of HO-1 in the huMSC-treated rats was achieved by huMSC activation of a MnSOD stimulation pathway.

It is possible that other, as yet unknown, factors are involved in kidney healing related to cells obtained from young animals, although Klotho is certainly a significant component of this pathway. Many studies have shown that low Klotho expression plays a role in the development of renal dysfunction [12–14]. In addition, Klotho deficiency promotes high inflammatory and pro-oxidative states [15, 16]. Klotho is predominantly known as an anti-ageing factor, and there is some evidence that its anti-ageing effects are achieved by downregulation of ROS signalling pathways. Furthermore, low levels of ROS-related stress have been demonstrated to extend the lifespan of a cell [19].

Oxidative stress can also be modulated by miRs, such as miR-34a and miR-335, which can promote a senescent profile in mesangial cells obtained from young animals, by antioxidant inhibition [19, 29]. MnSOD is a potential target of miR-335, and thioredoxin reductase 2, another antioxidant gene, is a potential target of miR-34a [19]. In addition, miR-34a regulates the expression of silent information regulator 1 (SIRT1), which increases the levels of cell-cycle inhibitor proteins, such as p21 [30], and miR-34a has also been reported to increase with ageing [31, 32], as well as in chronic inflammatory states [33]. Klotho deficiency and normal ageing may be associated with upregulation of miR-29a and miR-29b [34], and miR-29a is associated with inflammation as well [33]. Members of the miR-29 family suppress the protein phosphatase PPM1D, increasing apoptosis [30], and may function as markers of senescence because they can reduce the levels of type IV collagen, potentially weakening the basal membrane in senescent tissues [28]. In the present study, miR-29a and miR-34a were upregulated after IRI, and they were both downregulated by huMSC treatment.

Pro-oxidative pathways may induce nuclear factor kappa B signalling and inflammation [35]. In the present study, we showed that although the kidneys present few resident macrophages under normal conditions, macrophage numbers increase markedly after IRI [36]. In other rat studies, systemic macrophage depletion has been shown to attenuate renal IRI [37], and macrophage infiltration of the kidneys of rats has been found to increase renal fibrosis during the repair phase [38], possibly due to secretion of TGF-β. Therefore, less macrophage infiltration might translate into less fibrosis. In the acute phase, rats treated with huMSCs expressed less renal TGF-β1 and presented less chronic kidney damage (after 7 weeks) than did untreated rats, as demonstrated by biochemical and histological parameters.

On D2 the kidney tissue of the IRI rats showed very few lymphocytes. However, on D7 there were significant increases in CD3^+^ lymphocyte counts in IRI and IRI + huMSC rats. This corroborates the findings of other authors, who have shown that chemokine expression, a determinant of leukocyte migration, is increased during the repair phase that occurs approximately 7 days after IRI [39].

Production of ROS leads to premature senescence, as evidenced by the presence of β-gal as well as by upregulation of p16 and p21 [40]. Accordingly, the rats with ischaemia/reperfusion-induced AKI evaluated in the present study showed increased expression of these pro-senescence proteins, although this effect was reversed by treatment with huMSCs. It has been reported that AKI is associated with overexpression of p16 and p21 in kidney cells [41]. In addition, telomere-independent mechanisms of premature senescence have been shown to be associated with cell-cycle inhibitors [42]. Overexpression of p21 induces renal expression of β-gal, a marker of increased autophagy in senescent cells [43], and that expression has been shown to increase immediately after IRI [44]. Furthermore, overexpression of p21 and senescence can both induce TGF-β production, ultimately generating fibrosis in either case [45, 46].

Studies in p21^−/−^ mice have shown that more cell cycle activity and an increased number of PCNA-positive cells after AKI may be associated with impaired kidney function and increased mortality [20]. Given our finding that p21 expression was lower in IRI + huMSC rats than in IRI rats, the expectation was that there would have been greater tubular proliferation in the former group, which was not the case. However, in tumour studies Klotho has been shown to have antiproliferative effects, decreasing PCNA expression in colon cancer cells [47], and we hypothesise it may be protective during the acute phase of IRI.

Cell senescence can occur in a telomere-dependent (replicative) or telomere-independent (stress-induced) manner. Stress-induced senescence can be triggered by DNA damage, inflammatory processes or oxidative stress [48]. In the present study, we have demonstrated that AKI induction of senescence is a telomere-independent process, and that treatment with huMSCs protects kidneys from dysfunction and from oxidative stress-induced premature senescence after IRI.

## Conclusion

Our data indicate that treatment with young stromal cells attenuates the inflammatory and oxidative stress responses to ischaemia/reperfusion-induced AKI, as well as reducing the expression of senescence-related proteins and miRs. Our findings broaden perspectives for the treatment and prevention of maladaptive repair of AKI.

## Additional file

Additional file 1: Figure S1. showing telomerase activity determination. **A** Assay that combines PCR and ELISA techniques, by mixing total kidney protein with Taq DNA polymerase, nucleotides and two primers (one biotinylated TTAGGG primer, working as a telomerase substrate, and one anti-sense primer to amplify the reaction). **B** The amplified biotinylated sequences were then hybridised to a digoxigenin-labelled telomeric-specific detection probe and immobilised in a streptavidin-coated microplate. **C** The immobilised PCR product was detected with an antibody against digoxigenin, which was conjugated to peroxidase. **D** When the reaction occurs, it forms a coloured reaction product. (TIF 9425 kb)

## Abbreviations

AKI: Acute kidney injury
aMSC: Adipose-derived mesenchymal stromal cell
AQP2: Aquaporin 2
D2: Post-IRI day 2
D49: Post-IRI day 49
D7: Post-IRI day 7
FENa: Fractional excretion of sodium
HO-1: Heme oxygenase-1
huMSC: Human umbilical cord-derived mesenchymal stromal cell
IRI: Ischaemia/reperfusion injury
miR: microRNA
MnSOD: Manganese superoxide dismutase
MSC: Mesenchymal stromal cell
PCNA: Proliferating cell nuclear antigen
qPCR: Quantitative polymerase chain reaction
ROS: Reactive oxygen species
TRF: Terminal restriction fragment
β-gal: β-galactosidase
