# Supplementary figures and images for: Human umbilical cord-derived mesenchymal stromal cells protect against premature renal senescence resulting from oxidative stress in rats with acute kidney injury
Source: Stem Cell Res Ther. 2017 Jan 28;8:19. doi: 10.1186/s13287-017-0475-8 (PMC5273809; doi:10.1186/s13287-017-0475-8)

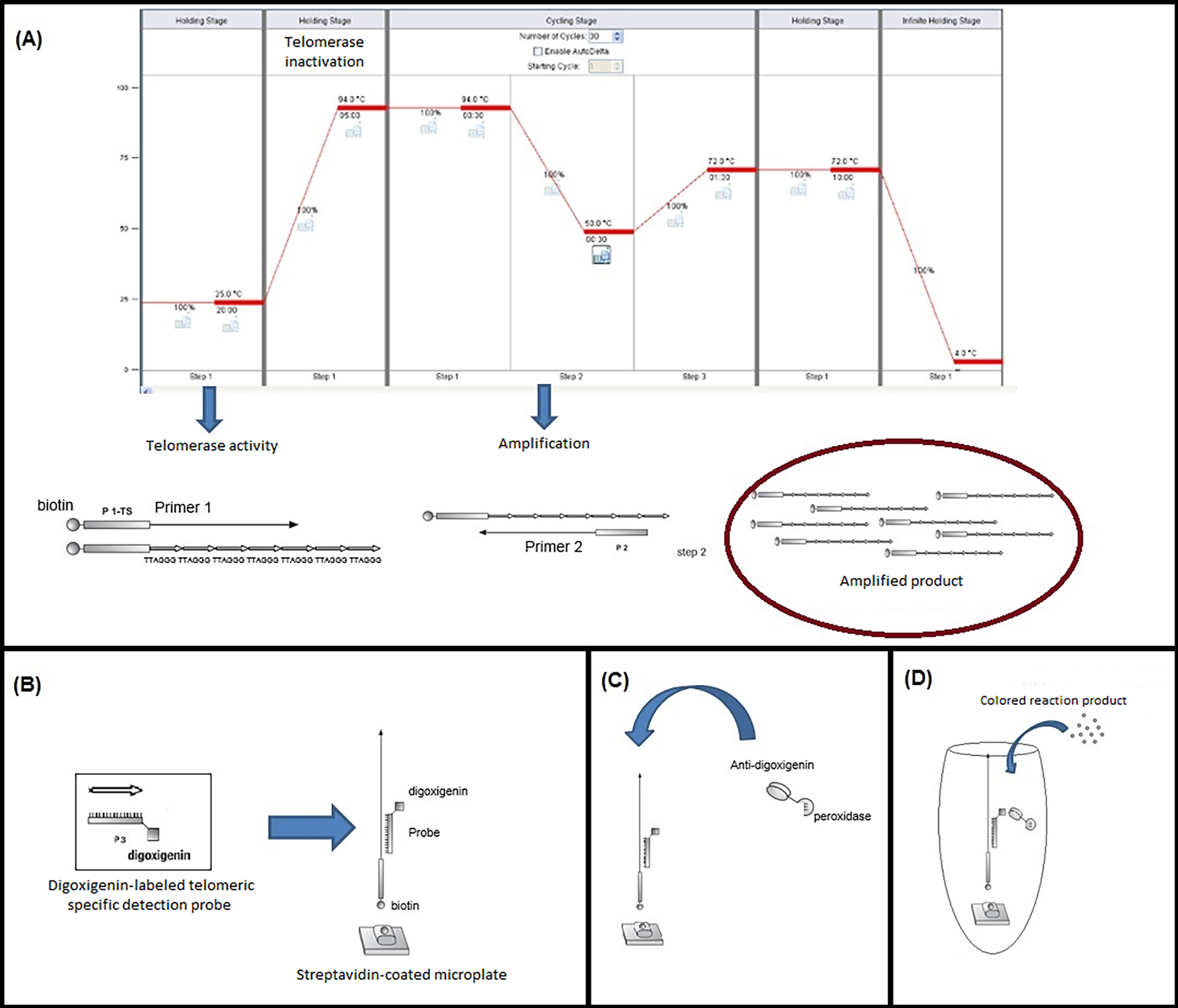

Supplement: Additional file 1: Figure S1. — showing telomerase activity determination. A Assay that combines PCR and ELISA techniques, by mixing total kidney protein with Taq DNA polymerase, nucleotides and two primers (one biotinylated TTAGGG primer, working as a telomerase substrate, and one anti-sense primer to amplify the reaction). B The amplified biotinylated sequences were then hybridised to a digoxigenin-labelled telomeric-specific detection probe and immobilised in a streptavidin-coated microplate. C The immobilised PCR product was detected with an antibody against digoxigenin, which was conjugated to peroxidase. D When the reaction occurs, it forms a coloured reaction product. (TIF 9425 kb) [file 13287_2017_475_MOESM1_ESM.tif]
